# Increased Expression of Tetraspanins, ALIX and HSP-70 in the Placenta Tissue of Women with Chronic Venous Disease during Pregnancy

**DOI:** 10.7150/ijms.87830

**Published:** 2023-10-16

**Authors:** Miguel A Ortega, María Asunción Sánchez-Gil, Oscar Fraile-Martínez, Cielo García-Montero, Luis G Guijarro, Raúl Diaz-Pedrero, Laura Lopez-Gonzalez, Juan De Leon-Luis, Coral Bravo, Melchor Álvarez-Mon, Julia Bujan, Miguel A Saez, Natalio García-Honduvilla

**Affiliations:** 1 Department of Medicine and Medical Specialities, Faculty of Medicine and Health Sciences, University of Alcalá, Alcala de Henares, Spain.; 2 Ramón y Cajal Institute of Sanitary Research (IRYCIS), Madrid, Spain.; 3 University Defense Center of Madrid (CUD), Madrid, Spain.; 4 Unit of Biochemistry and Molecular Biology, Centro de Investigación Biomédica en Red en El Área Temática de Enfermedades Hepáticas (CIBEREHD), Department of System Biology, University of Alcalá, Alcala de Henares, Spain.; 5 Department of General and Digestive Surgery, University Hospital Príncipe de Asturias, 28805 Madrid, Spain.; 6 Department of Surgery, Medical and Social Sciences, Faculty of Medicine and Health Sciences, University of Alcalá, 28801 Alcala de Henares, Spain.; 7 Department of Obstetrics and Gynecology, University Hospital Gregorio Marañón, Madrid, Spain.; 8 Health Research Institute Gregorio Marañón, Madrid, Spain.; 9 Department of Public and Maternal and Child Health, School of Medicine, Complutense University of Ma-drid, Madrid, Spain.; 10 Immune System Diseases-Rheumatology, Oncology Service an Internal Medicine, Centro de Investigación Biomédica en Red en El Área Temática de Enfermedades Hepáticas (CIBEREHD), University Hospital Príncipe de Asturias, Alcala de Henares, Spain.; 11 Pathological Anatomy Service, Central University Hospital of Defence-UAH, 28047 Madrid, Spain.

**Keywords:** Chronic venous disease (CVD), Pregnancy, placenta, Tetraspanins, ALG-2 interacting protein (ALIX), Heat shock protein 70 (HSP-70)

## Abstract

Chronic venous disease (CVD) is a complex and common vascular disorder characterized by increased blood pressure and morpho-functional changes in the venous system like varicose veins. Pregnancy is one of the main risk factors for suffering from this condition. Despite the consequences of CVD during pregnancy remains to be fully understood, compelling evidence support that this condition represents an important stress for the mother and the fetus, leading to significant histopathological changes in the placenta. Tetraspanins (CD9, CD63, and CD81), ALG-2-interacting protein X (Alix), and heat-shock protein (HSP-70) are cellular components involved in multiple biological processes under homeostatic and disease conditions. Despite some studies that have evidence of their relevance in the placenta tissue and pathological pregnancies, there is limited knowledge regarding their role in pregnancy-associated CVD. In this sense, the present work aims to analyze gene and protein expression of these components in the placenta of women with CVD (n=62) in comparison to healthy women (n=52) through RT-qPCR and immunohistochemistry, respectively. Our results show an increased gene and protein expression of the different studied markers, suggesting their potential involvement in the pathological environment of the placenta of women who undergo CVD during pregnancy. In this sense, further studies should be directed to deep into the potential implications of these changes to understand the effects and consequences of this condition in maternofetal wellbeing.

## 1. Introduction

Chronic venous disease (CVD) is a complex vascular disorder involving a profound remodeling of the venous system responsible for ambulatory venous hypertension, being varicose veins (VVs) its most common manifestation 1,2. CVD affects an important percentage of the population in Westernized societies, representing a notable socioeconomic burden, especially in its advanced stages 3. Pregnancy is one of the main risk factors related to CVD, and according to epidemiological data, approximately 1 in 3 women might present signs of CVD during pregnancy, while the risk of suffering from this condition increases progressively with the number of pregnancies 4. CVD in pregnancy is related to various hormonal, mechanical, and hemodynamical changes that occur in this period. In more detail, mechanical compression of the enlarged uterus with consequent hypertension in the lower limb veins, distension, and valve damage, seems to obstruct pelvic venous outflow, which together with the role of pregnancy-related hormones and the development of the fetus promotes the onset of CVD 5-8.

Precise clinical and biological consequences of CVD during pregnancy remain to be fully described. Up to date, it is known that suffering from this condition in pregnancy is associated with intrapartum fetal compromise 9 and a proinflammatory switch on circulating cytokines and chemokines in both pregnant women and their newborns 10, along with evidence of damage and compromise in maternofetal structures 11. The placenta seems to be an organ particularly affected during CVD, presenting significant functional and structural alterations 12,13. The placenta fulfills multiple roles during pregnancy, playing a key role in the maternofetal exchange and communication 14-16. Thus, deepening the pathophysiological implications of CVD in the placenta is essential to gain further insights into the role of this condition in maternofetal well-being.

Tetraspanins (CD9, CD63 and CD81) are a family of proteins with pleiotropic functions in cell biology and physiology. For instance, it has been described its role in regulating the function and/or trafficking of their partner proteins at the cell membrane, as well as in immunomodulation or as important markers of extracellular vesicles (EVs) 17. ALG-2-interacting protein X (Alix) is another protein involved in the modulation of several biological processes such as apoptosis endocytic membrane trafficking and cell adhesion, whereas the chaperone heat-shock protein (HSP-70) also participates in a wide range of homeostatic processes by controlling proteostasis 18,19. An altered detection of tetraspanins ALIX, or HSP-70 has been demonstrated in maternofetal structures and extracellular vesicles of women under pathological pregnancies 20-22. However, there is a lack of studies evaluating the expression of these markers in the placenta of women with CVD. Therefore, the present study aims to evaluate gene and protein expression of tetraspanins, ALIX, and HSP-70 in the placentas of women with CVD by performing real-time quantitative PCR (RT-qPCR) and immunohistochemistry (IHC), respectively.

## 2. Patients and methods

### 2.1 Experimental design

In the current research, 114 pregnant women in their third trimester participated in an observational, analytical, prospective study. 62 women with a clinical diagnosis of CVD according to the CEAP classification [23]and 52 women without a history of CVD (referred to as HC) were included. The median age for the CVD group was 33 years old, and the interquartile range (IQR) was 22-40 years. In parallel, the median age for the HC group was 34 (IQR, 27-41 years). For women with CVD, the median gestational period was 40.5 weeks (with an IQR of 39-41.5 weeks), and for women with HC, it was 41 weeks. (IQR, 39-42 weeks).

During the third-trimester consultation, each woman's clinical history was updated, and physical examinations were finished. In order to examine ultrasound pictures of the lower extremities, Eco-Doppler (Portable M-Turbo Eco-Doppler; SonoSite, Inc., Bothell, WA, USA) operating at 7.5 MHz was employed. Our study included women over the age of 18 who had CVD (CEAP 1) in their lower limbs during the third trimester. Women who had previously displayed any of the conditions listed below were not included:: (1) arterial hypertension; (2) venous malformations; (3) heart failure; (4) BMI of less than 25; (5) clinical diagnosis of type 1/type 2/gestational diabetes mellitus or other endocrine diseases; (6) autoimmune diseases; (7) active infectious processes; (8) Preeclampsia and/or HELLP syndrome, (9) fetal growth restriction from known causes, (10) placental infarction, (11) avascular villi, (12) delayed villus maturation, (13) chronic villitis, (14) habitual alcohol, tobacco, or drug use (1 unit/day), (15) evidence of cardiovascular disease before pregnancy, and (16) the emergence of any exclusion criteria up until delivery.

Each patient received the necessary informed consent before enrollment, giving their signed, written permission. This work was carried out following the ethical principles of autonomy, beneficence, non-maleficence, and distributive justice, as well as the rules of good clinical practice, the principles of the Declaration of Helsinki (2013), and the Oviedo Convention (1997), and it was approved by the Clinical Research Ethics Committee of the Central University Hospital of Defense University of Alcalá (03-37/17 on 3 March 2017).

### 2.2 Tissue Collection and Processing

Postpartum placental biopsies were given by all 114 subjects. A scalpel was used to cut five pieces of placenta from each patient's sample, each of which included a variety of mixed cotyledons. They were then divided into two sterile tubes, one containing RNAlater® solution (Ambion; Thermo Fisher Scientific, Inc., Waltham, MA, USA), and the other containing minimal essential medium (MEM; Thermo Fisher Scientific, Inc., Waltham, MA, USA) with 1% antibiotic/antimycotic (streptomycin, amphotericin B, and penicillin; Thermo Fisher Scientific, Inc.). After that, the samples were processed in a class II laminar flow hood (Telstar AV 30/70 Müller 220 V 50 MHz; Telstar; Azbil Corporation) under sterile conditions. Following that, samples were stored to be processed later for a study of gene expression in 1 mL of RNAlater® at -80 °C.

After that, erythrocytes were removed by washing and rehydrating preserved MEM samples five times in antibiotic-free MEM. Following that, they were cut into 2 cm-long pieces with a scalpel and fixed in F13 (a solution made up of 60% ethanol, 20% methanol, 7% polyethylene glycol, and 13% distilled water), following procedures that have been gathered 12. Molds were then used to create paraffin-embedded samples. We used an HM 350 S rotary microtome to cut slices that were 5 µm thick after the paraffin had solidified (Thermo Fisher Scientific, Inc., Waltham, MA, USA). They were then gathered on glass slides that had been treated with 10% polylysine to aid in the sections' adhesion after being stretched in a hot water bath.

### 2.3 Studies on Gene Expression Using Quantitative PCR and reverse transcription (RT-qPCR)

As reported in earlier studies 24, we first extracted RNA using the guanidinium thiocyanate-phenol-chloroform method. The mRNA expression amounts of particular genes can be studied using this technique.

Reverse transcription (RT) was used to create complementary DNA (cDNA) from 50 ng/L of RNA samples. Each sample was combined with 4 µL of 0.25 µg/ µL oligo-dT solution (Thermo Fisher Scientific, Inc., Waltham, MA, USA) and then put in an AccuBlock dry bath (Labnet International Inc., Edison, NJ, USA) at 65 °C for 10 minutes to cause RNA denaturation. Following that, 10 µL of a reverse transcription mix comprising the following items was added to the samples on ice.

Reverse transcription was done using a G-Storm GS1 thermocycler (G-Storm Ltd., Middlesbrough, UK). The samples were then heated for one hour and fifteen minutes at 37 °C to encourage cDNA synthesis. The reverse transcriptase was then denaturized by raising the temperature to 70 °C and holding it there for 15 minutes. The temperature was thereafter gradually lowered to 4 °C. Negative reverse transcription was equally performed in RNA samples where the M-MLV RT enzyme was swapped out for DNase- and RNase-free water to ensure the lack of genomic DNA contamination. When not in use, cDNA produced at room temperature was diluted 1:20 in water free of DNase and RNase.

Through the Primer-BLAST and AutoDimer online tools, specific primers for the chosen genes (Table 1) were designed de novo 25,26. To standardize the findings, the constitutively expressed TATA-box binding protein (TBP) gene was used as a control 27. The proportional amounts of mRNA are used to express the gene expression units. The relative standard curve technique was used to conduct RT-qPCR on a StepOnePlus^TM^ System (Applied Biosystems; Thermo Fisher Scientific, Inc.). The following was the outcome of the reaction: Forward and reverse primers, as well as 3 µL of DNase and RNase-free water, were combined with 5 µL of material and 10 µL of iQTM SYBR® Green Supermix (Bio-Rad Laboratories, Inc. Hercules, California, USA) at a ratio of 1:20 before being added to a MicroAmp® 96-well plate. (Applied Biosystems; Thermo Fisher Scientific, Inc., Waltham, MA, USA). The following thermocycling conditions were used: initial denaturation for 10 minutes at 95 °C, denaturation for 15 seconds at 95 °C, annealing at different temperatures depending on the melting temperature of each primer combination for 30 seconds, and elongation at 72 °C for 1 minute, for 40 to 45 cycles. Then, a dissociation curve was created for 15 seconds at 95 °C, 1 minute at 60 °C, 15 seconds at 95 °C, and 15 seconds at 60 °C. After each repetition cycle (amplification), as well as at various points along the dissociation curve, fluorescence was detected. The information gathered from the aforementioned genes was included in a standard curve that was created through serial dilutions of a mixture of samples that were added to each plate by the constitutive expression of TBP (as per the manufacturer's procedures). All placenta tissue samples underwent two rounds of this RT-qPCR.

### 2.4 Immunohistochemical analysis

For the detection of antigen/antibody interactions, avidin-biotin complexes with avidin peroxidase were used 13. Placenta samples embedded in paraffin underwent immunohistochemical investigations. The protocol requirements include information about the antibodies used (Table 2). The primary antibody was incubated with placental samples for 1.5 hours. It was then treated with 3% BSA Blocker and PBS at 4 °C overnight (cat. no. 37,525; Thermo Fisher Scientific, Inc., Waltham, MA, USA). The following day, placental samples were incubated for 1.5 hours at room temperature with a biotin-conjugated secondary antibody that had earlier been diluted in PBS. ExtrAvidin®-Peroxidase (Sigma-Aldrich; Merck KGaA, San Luis, MO, USA) was then added and allowed to sit at room temperature for an additional hour (1:200 dilution in PBS). The protein expression was lastly assessed using chromogenic diaminobenzidine (DAB) substrate kit (cat. no. SK-4100; Maravai LifeSciences, San Diego, CA, USA). This kit was prepared immediately before (5 mL distilled water; four drops of DAB; two drops of hydrogen peroxide and two drops of buffer).

The signal was detectable as dark stains after using the peroxidase chromogenic substrate for 15 min at room temperature. Negative controls for the various proteins were given to each section, swapping out the primary antibody incubation for a PBS solution. To produce contrast in all tissues, Carazzi hematoxylin was applied for 15 minutes.

For each patient in the HC and CVD groups, a total of 10 areas of view in 5 distinct sections were examined at random. According to the immunoreactive score (IRS), which was established in earlier research 28,29, a patient was classified as positive if the stained mean area in the studied sample was less than 5% of the total. Two independent histologists assessed the immunostaining, and each sample was then graded according to the following scale: In order from 0 to 1, there is minimal staining (25%), intermediate staining (25-65%), and strong staining (65-100%). The tissue analysis was done using a Zeiss Axiophot optical microscope (Carl Zeiss, Oberkochen, Germany).

### 2.5 Statistical Analysis

The GraphPad Prism® v6.0 software (GraphPad, Inc., San Diego, CA, USA) was used to process the statistical data. The median and standard error of the mean (SEM) are used in the Mann-Whitney U test to compare the two groups. The thresholds for significance were set at p<0.05 (*), p<0.01 (**), and p<0.001 (***).

## 3. Results

### 3.1 Women with chronic venous disease during pregnancy exhibit increased expression of tetraspanins (CD9, CD63, and CD81) in the placenta

**Figure 1:**
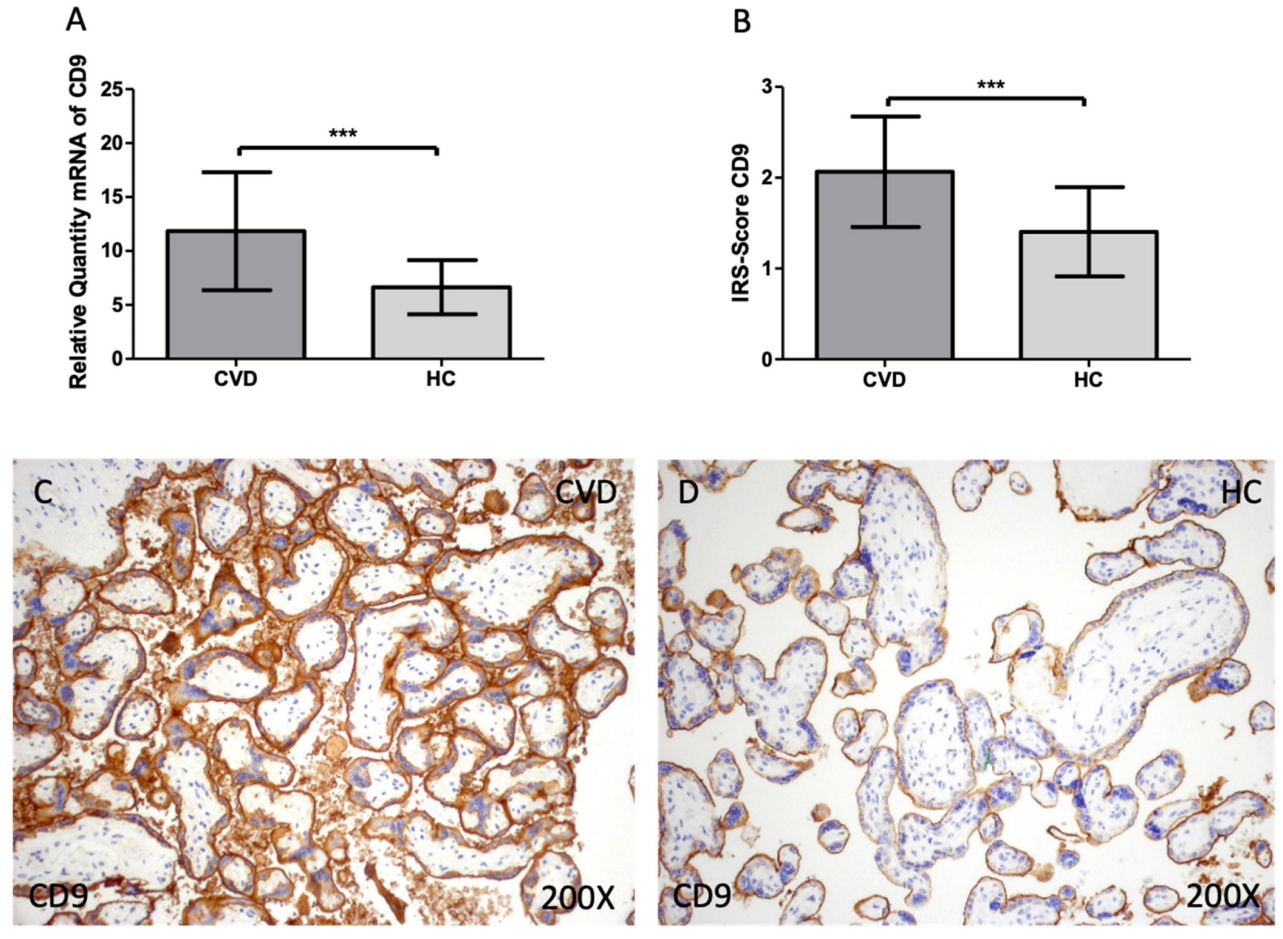
(A). CD9 mRNA expression in the HC group (healthy controls) and CVD patients (chronic venous disease) (B). Protein expression of CD9 based on IRS-Scores in the placenta villi of the HC and CVD group. (C, D). Images showing the immunostaining for CD9 in the placenta villi of the CVD group and the HC group. p < 0.001 (***); p<0.01 (**); p<0.05 (*).

Firstly, we evaluated the gene and protein expression of tetraspanins (CD9, CD63 and CD81) in the placenta of women with CVD. According to our findings, there is a statistically significant increase in CD9 gene expression in the placental tissue of pregnant women who have CVD [Figure 1A; CVD = 11.838 ± 5.466, HC = 6.641 ± 2.509, ***p=0.0003] The placental villi of women with CVD displayed a significant rise in protein expression of CD9, according to histological analysis [Figure 1B; CVD = 2.065 ± 0.609, HC = 1.405± 0.490, ***p=0.0007]. Regarding histopathological images, women with CVD had significantly more CD9 protein expression throughout the placental villi [Figure 1C, D].

**Figure 2:**
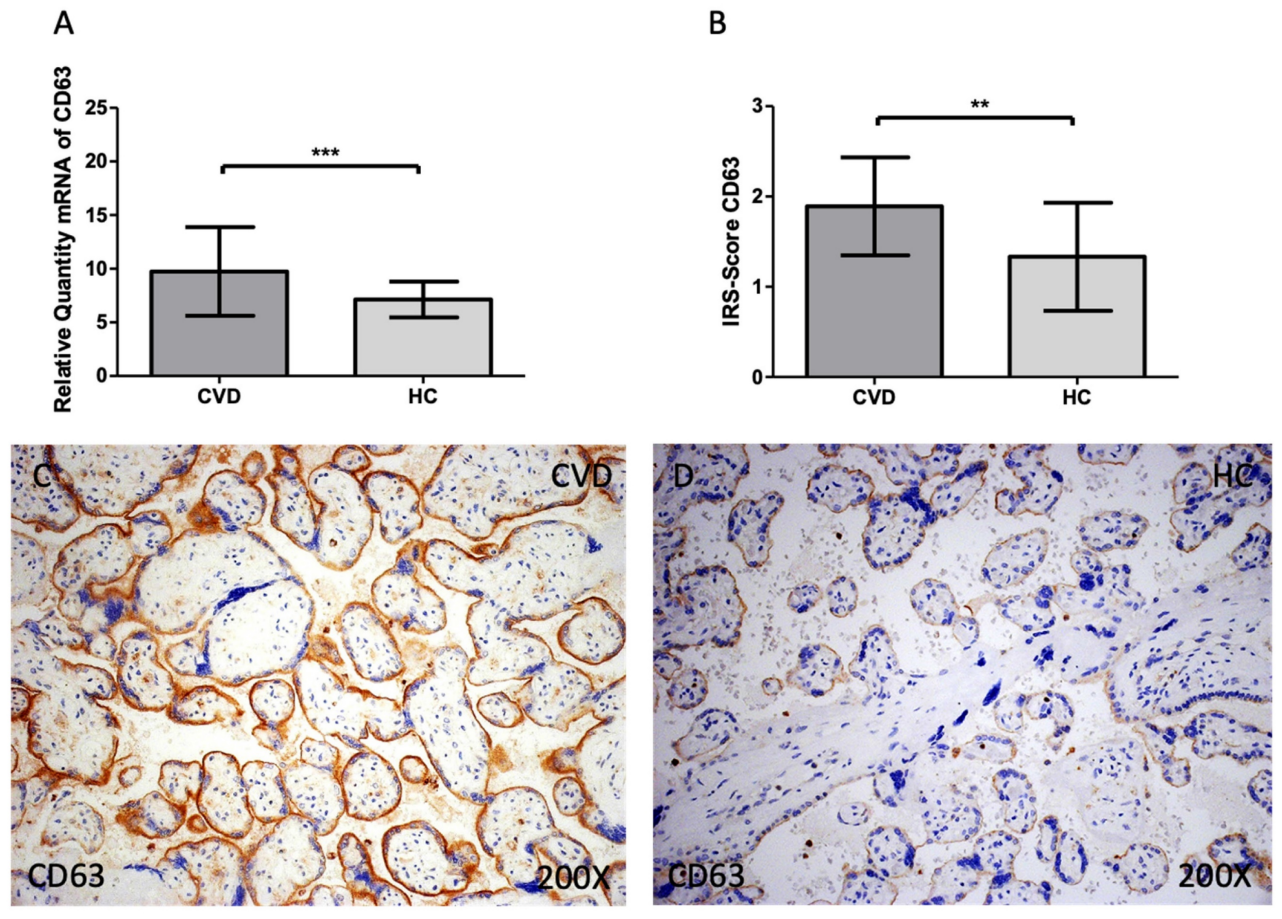
(A). CD63 mRNA expression in the HC group (healthy controls) and CVD patients (chronic venous disease) (B). Protein expression of CD63 based on IRS-Scores in the placenta villi of the HC and CVD group. (C, D). Images showing the immunostaining for CD63 in the placenta villi of the CVD group and the HC group. p < 0.001 (***); p<0.01 (**); p<0.05 (*).

When compared to HC, women with CVD had significantly increased levels of CD63 gene expression in their maternal villi [Figure 2A; CVD = 9.742 ± 4.137, HC = 7.130 ± 1.662, **p=0.0082]. When the placental villi of women with CVD were subjected to a histological analysis this significant decline was also defined [Figure 2B; CVD = 1.891 ± 0.543, HC = 1.333 ± 0.599, **p=0.0034]. Histological images show that the expression of CD63 is higher in the CVD group in the different structures of the placental villi when compared to HC [Figure 2C, D].

**Figure 3:**
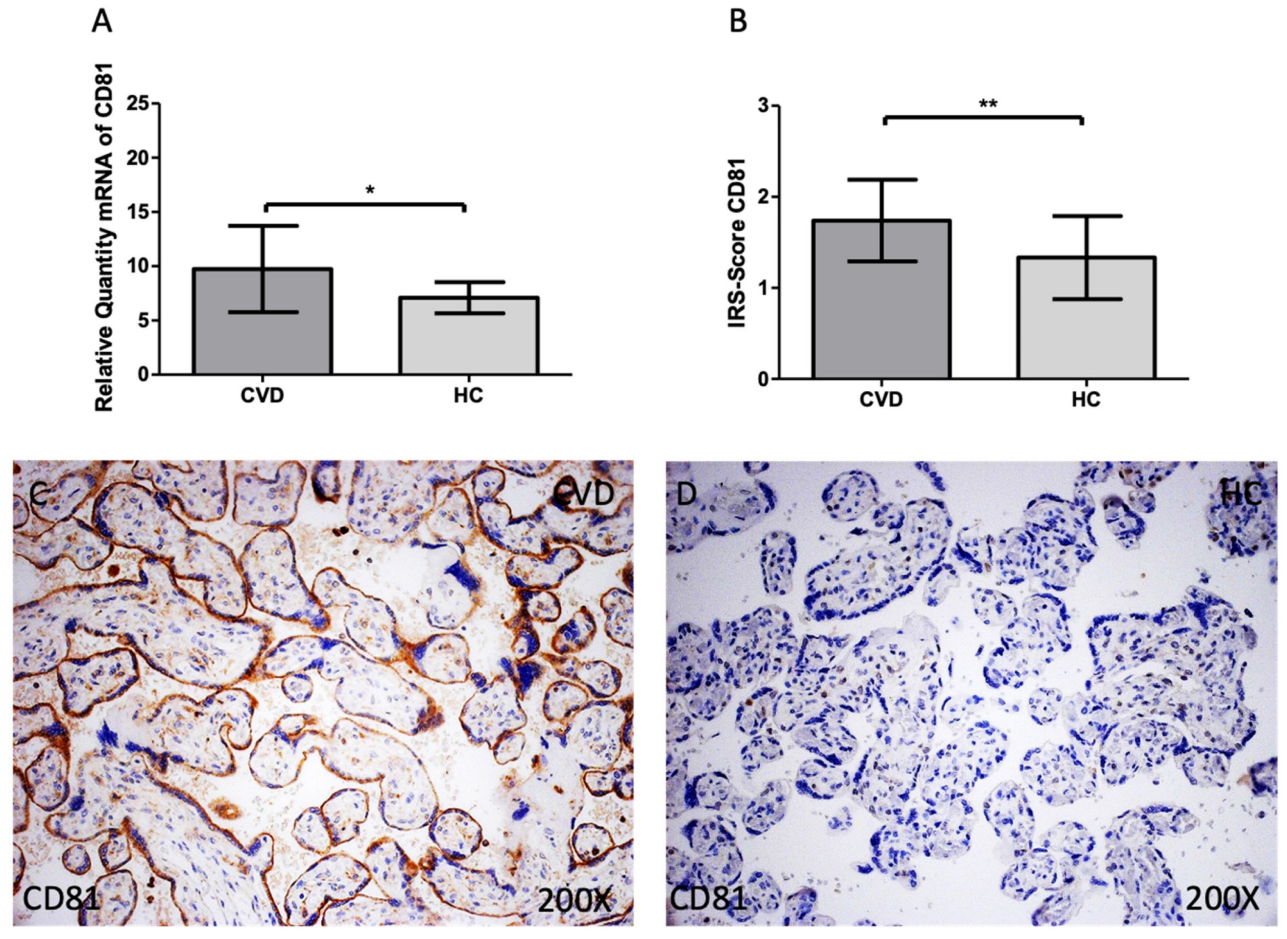
(A). CD81 mRNA expression in the HC group (healthy controls) and CVD patients (chronic venous disease) (B). Protein expression of CD81 based on IRS-Scores in the placenta villi of the HC and CVD group. (C, D). Images showing the immunostaining for CD81 in the placenta villi of the CVD group and the HC group. p < 0.001 (***); p<0.01 (**); p<0.05 (*).

The same tendency was observed for CD81 expression. We observed that the gene expression of this marker was significantly increased in the CVD group when compared to HC [Figure 3A; CVD = 9.739 ± 3.982, HC = 7.096 ± 1.443, *p=0.0213]. For protein expression, a significant increase of CD81 was also observed in the CVD group [Figure 3B; CVD = 1.739 ± 0.449, HC = 1.333 ± 0.456, **p=0.0074]. Histologically, the increased protein expression of this marker was reported in the placenta of women with CVD throughout the chorionic villi [Figure 3C, D].

### 3.2 Women with chronic venous disease during pregnancy present augmented expression of ALIX in the placental tissue

**Figure 4:**
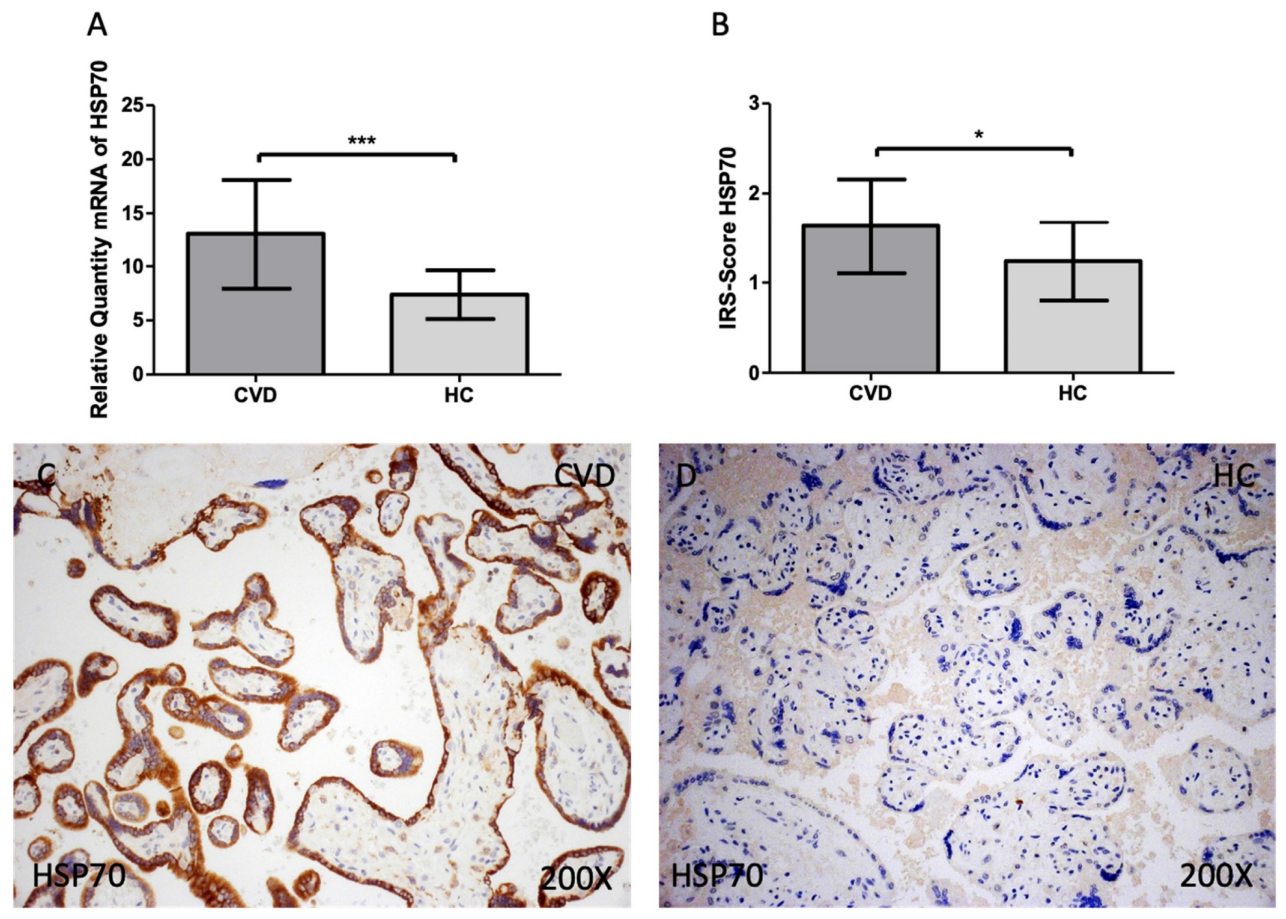
(A). ALIX mRNA expression in the HC group (healthy controls) and CVD patients (chronic venous disease) (B). Protein expression of ALIX based on IRS-Scores in the placenta villi of the HC and CVD group. (C, D). Images showing the immunostaining for ALIX in the placenta villi of the CVD group and the HC group. p < 0.001 (***); p<0.01 (**); p<0.05 (*).

In the analysis of gene and protein expression of ALIX, we report an increased gene expression of this marker in the CVD group when compared to HC [Figure 4A; CVD = 18.699 ± 3.509, HC = 8.080 ± 3.144, ***p<0.0001]. In the case of protein expression, a significant increase of ALIX was reported in the CVD group [Figure 4B; CVD = 2.565 ± 0.379, HC = 1.667 ± 0.577, ***p<0.0001]. Histologically, the increased protein expression of this marker was equally observed throughout the placental villi of women with CVD [Figure 4C, D].

### 3.3 Women with the chronic venous disease during pregnancy display greater gene and protein expression of HSP-70 in the placenta

**Figure 5:**
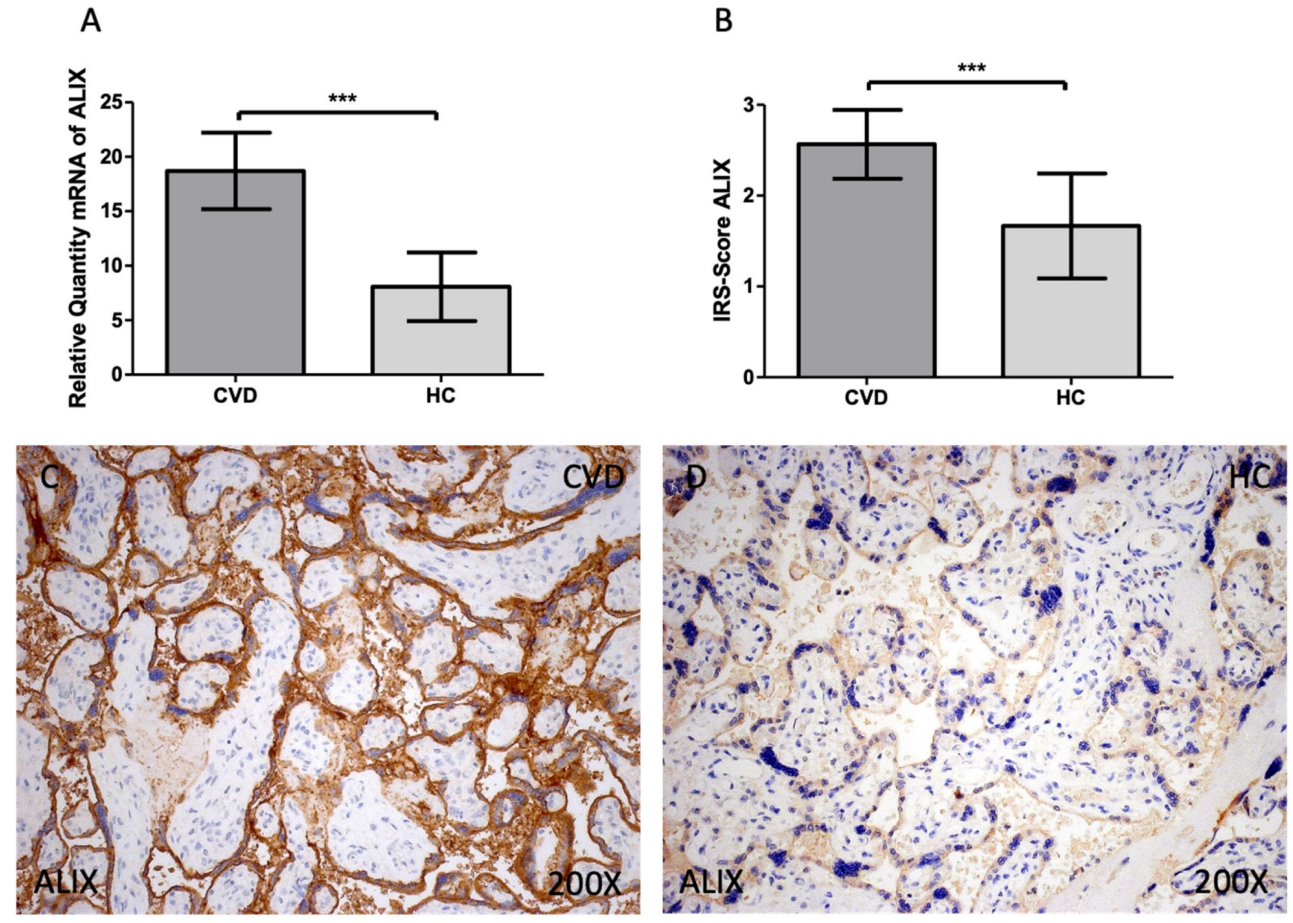
(A). HSP-70 mRNA expression in the HC group (healthy controls) and CVD patients (chronic venous disease) (B). Protein expression of HSP-70 based on IRS-Scores for in the placenta villi of the HC and CVD group. (C, D). Images showing the immunostaining for HSP-70 in the placenta villi of the CVD group and the HC group. p < 0.001 (***); p<0.01 (**); p<0.05 (*).

Finally, we observed a statistically significant increase in HSP-70 gene expression in the placental tissue of pregnant women who have CVD [Figure 5A; CVD = 13.007 ± 5.090, HC = 7.387 ± 2.251, ***p<0.0001] The placental villi of women with CVD displayed a significant rise in protein expression of HSP-70, according to histological analysis [Figure 5B; CVD = 1.630 ± 0.527, HC = 1.238 ± 0.436, *p=0.0175]. Regarding histopathological images, women with CVD had significantly higher HSP-70 protein expression throughout the placental villi [Figure 5C, D].

## 4. Discussion

The placenta is a complex tissue with pleiotropic functions during gestation. This organ is crucial for ensuring pregnancy success, whereas alterations in this structure are tightly linked to different obstetric complications, including gestational CVD 14 in which several morpho-functional alterations have been described, with detrimental consequences for both the mother and fetus 9,10,13. In this sense, our study reveals a possible pathophysiological role of an increased tissue and gene expression of tetraspanins (CD9, CD63 and CD81), ALIX and HSP-70 in the placenta of women affected by gestational CVD, aiding the potential implications of CVD during pregnancy.

Tetraspanins are a family of proteins with pleiotropic functions in cell biology and physiology. These molecules associate specifically and directly with a limited number of proteins, and also with other tetraspanins, generating a complex network of interactions involved in cell and membrane compartmentalization 17. Tetraspanins play a critical role in extracellular vesicle biogenesis, cargo selection, cell targeting, and cell uptake under both physiological and pathological obstetric conditions 30. CD9, CD63 and CD81 are especially enriched in the membrane of exosomes, being commonly used as exosome biomarkers 30-32. Compelling evidence supports the relevance of exosomes and other extracellular vesicles in physiological and pathological pregnancies, especially those derived from the placenta 33. However, to date, there are no studies evaluating the relevance of placental-derived extracellular vesicles in women with CVD. An increased expression of tetraspanins might be used to encourage further studies in the quantification and characterization of extracellular vesicles in these patients, due to the multiple implications and translational applications derived from this field 34,35. On the other hand, CD9, CD63, and CD81 have a broad tissue distribution, and they are commonly found in plasma membrane. For this reason, despite being generally accepted as exosome markers, in certain cases, they cannot be used to discriminate them from other types of extracellular vesicles like microvesicles 31. In this sense, our study reports an increase of classical extracellular vesicle markers, probably related to exosomes but certainly originated from the placenta of women suffering from CVD, as shown by histopathological techniques. CD9, CD63 and CD81 have been shown to play an important role in the placenta under physiological and pathological conditions, especially when found at extracellular vesicles 30,36. For instance, CD9 seems to be essential in the process of trophoblast adhesion and invasion 37,38, whereas CD63 seems to exert some notable immunomodulatory functions 39. Concomitantly, an overexpression of CD81 promotes pathological changes that occur in both the placenta and maternal endothelial cells, inducing a pre-eclampsia-like phenotype in pregnant rats 22*.* The role of these markers in the placenta of women with CVD has not been established yet. But their increased presence may be an indicator of an increased exosome and extracellular vesicle production by the placenta in these patients, although further studies in this sense are strongly warranted.

Alix is an accessory protein of the endosomal sorting complex required for transport (ESCRT), participating in several cellular functions such as endosome sorting, membrane fission, viral budding, cytokinesis, and apoptosis 40. The interaction of ALIX with ESCRT makes this protein a major regulator of the biogenesis of extracellular vesicles and for determining their cargo 41-43. The validation of ALIX as a marker of extracellular vesicles derived from the placenta has been demonstrated in previous works 33,44,45. Apart from its relevance as an extracellular vesicle marker, it is of note that ALIX can be involved in the process of apoptosis due to its modulatory role on apoptosis-linked gene 2 (ALG-2) -a calcium-binding protein necessary for cell death- and other mechanisms 46. Prior studies have evidenced that the placental villi of women with CVD present enhanced apoptosis in comparison to non-pathological pregnancies 47. Thus, it is likely that a higher expression of ALIX may be implicated in the apoptotic events that occurred in this tissue related to CVD.

Finally, our study supports an increased HSP-70 expression in the placenta of women with CVD. HSP70 is engaged in several cellular functions, including protein folding, transport, and degradation 48. This protein is upregulated under stressful stimuli, such as heat, exercise, or pathological stress 49. HSP70 has been demonstrated to be found in extracellular vesicles like exosomes and microvesicles, which indicates that it may be involved in their biogenesis, secretion, and function 50. HSP70 association with extracellular vesicles occurs at high levels under disease conditions, although they can also be translocated to the cell membrane or secreted in a soluble form 51. Indeed, HSP70-containing extracellular vesicles have been linked to several normal and pathological processes, including immune system control or cancer development 52. An increased expression of HSP-70 has been found in the placenta of women with pre-eclampsia, placental vascular diseases, and other pathological pregnancies 21,53,54. An increased expression of cellular stress markers has been demonstrated in the placenta of women with CVD 55, Among the mechanisms of response orchestrated by HSP70 in maternofetal tissues, previous works have identified this protein as a prominent angiogenic factor 56. In this sense, increased angiogenesis and lymphangiogenesis seems to be reported in the placenta of women with CVD 29,57. Thus, our results suggest that the pathological environment of the placenta observed in women with CVD can be related to the enhanced HSP-70 expression in this tissue, also suggesting a potential translocation into extracellular vesicles that remains to be further explored.

Main limitations in our study include 1) the possibility of including a greater sample size to confirm our results or a broader number of markers related; 2) The need of correlating histopathological changes with possible clinical consequences observed in the mother and/or fetus; and 3) That we have not isolated and characterized extracellular vesicles in maternofetal fluids. Therefore, the possible presence, role and applications of placental-derived extracellular vesicles in women with CVD remains to be determined and proven.

## 5. Conclusions

In the present article, we evidence an increased gene and protein expression of tetraspanins (CD9, CD63 and CD81), ALIX and HSP-70 in the placenta of women with CVD. This work gains further insights into the pathophysiological signatures of this condition in pregnancy, as well as the potential relevance of these markers in the placenta behavior and function. Because of the association of these markers with extracellular vesicles and the relevant role that they have in normal and pathological pregnancies, future efforts should be placed to measure and characterize extracellular vesicles derived from the placenta in maternal and fetal blood in women affected by CVD during pregnancy.

### Author Contributions

All authors have read and agreed to the published version of the manuscript.

### Funding

This study has been funded by Instituto de Salud Carlos III (ISCIII) through the project "PI21/01244" and co-funded by the European Union, as well as P2022/BMD-7321 (Comunidad de Madrid) and ProACapital, Halekulani S.L. and MJR.

### Institutional Review Board Statement

The study was conducted according to the guidelines of the Declaration of Helsinki and approved by the Ethics Committee of Clinical Investigations of the Central University Hospital of Defense Gómez-Ulla-UAH (03-37/17 on 3 March 2017).

### Informed Consent Statement

Informed consent was obtained from all subjects involved in the study.

### Data Availability Statement

The data used to support the findings of the present study are available from the corresponding author upon request.

## Figures and Tables

**Table 1:** Primers used for RT-qPCR: sequences and binding temperatures (Temp).

GENE	SEQUENCE Fwd (5^´^→3^´^)	SEQUENCE Rev (5´→3´)	Temp
**TBP**	TGCACAGGAGCCAAGAGTGAA	CACATCACAGCTCCCCACCA	60ºC
**CD9**	CACCGTGAAGTCCTGTCCTG	CTAGACCATCTCGCGGTTCC	55^O^C
**CD63**	CAGCCTTGGGAAGCCCAG	AGCCCCCTGGATTATGGTCT	60^O^C
**CD81**	AATTTCGTCTTCTGGCTGGCT	CCCGTCTCCACTCATACACG	61^O^C
**ALIX**	TCCAGCAGACTTACCCAAGC	CAGGGAACACCTCCTGGAAATAA	60^O^C
**HSP-70**	AGCTGGAGCAGGTGTGTAAC	CAGCAATCTTGGAAAGGCCC	59^O^C

**Table 2:** Primary and secondary antibodies.

Antigen	Species	Dilution	Provider	Protocol Specifications
**Primary antibodies**				
**CD9**	Rabbit monoclonal	1:500	Abcam ( ab236630)	100% Triton 0.1% in PBS, 10 minutes, before incubation with blocking solution
**CD63**	Mouse monoclonal	1:1500	Abcam (ab1318)	100% Triton 0.1% in PBS, 10 minutes, before incubation with blocking solution
**CD81**	Mouse monoclonal	1:250	Abcam (ab79559)	EDTA pH=9 before incubation with blocking solution
**ALIX**	Rabbit polyclonal	1:100	Abcam (ab76608)	EDTA pH=9 before incubation with blocking solution
**HSP-70**	Mouse monoclonal	1:100	Abcam (ab2787)	EDTA pH=9 before incubation with blocking solution
**Secondary antibodies**				
**Rabbit IgG**	Mouse	1:1000	Sigma-Aldrich (RG-96/B5283)	------
**Mouse IgG**	Goat	1:300	Sigma-Aldrich (F2012/045K6072)	------
